# Prioritization of clinical questions for the Australian Living Guideline for the Pharmacological Management of Inflammatory Arthritis

**DOI:** 10.1111/1756-185X.14926

**Published:** 2023-09-23

**Authors:** Samuel L. Whittle, Vanessa Glennon, Rachelle Buchbinder

**Affiliations:** ^1^ School of Public Health and Preventive Medicine Monash University Melbourne Victoria Australia; ^2^ Rheumatology Unit Queen Elizabeth Hospital Adelaide South Australia Australia

**Keywords:** guidelines, living evidence, rheumatoid arthritis

## Abstract

**Aim:**

Living guidelines aim to reduce delays in translating new knowledge into practice by updating individual recommendations as soon as relevant new evidence emerges. We surveyed members of the Australian Rheumatology Association (ARA) to develop a list of priority questions for the Australian Living Guideline for the Pharmacological Management of Inflammatory Arthritis (ALG) and to explore clinicians' use of clinical practice guidelines.

**Methods:**

An electronic survey of ARA members was performed in two phases. The first survey contained questions about current guideline use and beliefs and invited participants to submit at least three questions relevant to the management of rheumatoid arthritis (RA). In the second round, participants selected 10 questions they considered to be the highest priority from the collated list and ranked them in priority order. The sum of ranks was used to generate a final priority list.

**Results:**

There were 115 (21%) and 78 (14%) responses to the first and second survey rounds respectively. 87% of respondents use existing rheumatology guidelines in their usual practice, primarily EULAR guidelines. Most respondents favored the development of Australian rheumatology guidelines. In total, 34 potential recommendation topics were identified and ranked in order of priority.

**Conclusion:**

A list of 34 clinical questions about RA management, ranked in order of importance by clinicians, has informed the development of the ALG. Similar prioritization exercises in other contexts may permit guidelines to be tailored to the needs of guideline users in their specific context, which may facilitate international collaboration and promote efficient translation of evidence to practice.

**Plain Language Summary**

- Clinical questions that are most suited to living guidelines are those for which there is uncertainty in the evidence, new evidence is emerging, and which are considered by clinicians to be the most important questions. However, few guidelines have surveyed potential guideline users to identify the clinical questions of the highest importance.
- As part of the development of the Australian Living Guideline for the Pharmacological Management of Inflammatory Arthritis, the first rheumatology guideline to use living evidence methodology, 34 potential recommendation topics were identified and ranked in order of priority by rheumatologists and rheumatology health professionals.
- Replication of the prioritization exercise in other healthcare settings may help to further define the questions most suited to international collaboration and permit resources to be directed to a global living evidence synthesis for the most important questions.

## 1 BACKGROUND

Clinical practice guidelines are an important tool for improving the translation of research into practice, reducing unwanted variation in care, and facilitating shared clinical decision‐making.^1^ The recent emergence of living evidence methodology has been a major advance in guideline development. Living systematic reviews are performed with the same methods and rigor as standard systematic literature reviews, but are designed a priori to enable ongoing continuous surveillance of the literature so that relevant new evidence can be incorporated and the review updated in near real‐time.^2^

Similarly, living guidelines are designed to be updated whenever relevant new evidence becomes available so that clinicians, consumers, and other guideline users can be confident that the guideline recommendations are based on a synthesis of all available evidence. A key difference from traditional “static” guidelines is that updates to living guidelines occur at the level of the individual recommendation, whenever new information relevant to the specific recommendation emerges, rather than the guideline as a whole.^3^ Furthermore, living methodology allows guidelines to be developed one recommendation at a time, as resources permit.

Recommendations that are particularly suited to a living approach focus on topics for which the evidence base remains uncertain, where new evidence is emerging, and that are considered to be of high clinical importance.^3^ A formal process for determining the questions of highest relevance to clinicians is an important step in developing a guideline that is best suited to a living approach, makes efficient use of scarce resources, and is likely to be of value in clinical practice.

A variety of methods for priority‐setting in guideline development have been used, although many have focused on identifying which guidelines to develop rather than individual recommendations within a guideline.^4^ A review of prioritization exercises used in the development of new health practice guidelines found that most focused on guideline topics rather than specific clinical questions.^5^ A focus on the questions of highest importance to clinicians has been used to develop high‐impact international rheumatology guidelines by the 3e (Evidence, Expertise, Exchange) Initiative on topics including gout,^6^ undifferentiated peripheral inflammatory arthritis,^7^ and pain management in inflammatory arthritis.^8^

The Australia and New Zealand Musculoskeletal (ANZMUSC) Clinical Trials Network, in conjunction with the Australian Rheumatology Association (ARA), has commenced the development of an Australian Living Guideline for the Pharmacological Management of Inflammatory Arthritis (ALG).^9^ The ALG is the world's first rheumatology clinical guideline to be developed using the living methodology. It incorporates living systematic literature reviews and GRADE methodology to develop recommendations that are based on the latest evidence and are tailored to the Australian rheumatology context.^10^ Involvement of guideline users from the start of the guideline development process increases the relevance of the recommendations to local practice needs and may improve the translation of evidence into practice.^11^

In order to facilitate the development of the ALG, we undertook a survey of members of the ARA with two aims: (1) To systematically identify and rank in order of priority a set of clinical questions regarding the pharmacological management of rheumatoid arthritis (RA) and (2) To identify Australian rheumatology clinicians' attitudes toward clinical practice guidelines, including their current use, potential barriers, and opinions on the need for an Australian rheumatology guideline.

## 2 MATERIALS AND METHODS

### 2.1 Design and participants

The prioritization procedure consisted of two consecutive electronic surveys of members of the ARA. The process was designed to incorporate some of the features of the Delphi methodology, including anonymous participation and iteration of responses based on summary and feedback, in order to generate a consensus output that is not dominated by an individual or small group of participants.^12^ The ARA is the professional association for rheumatologists, rheumatology trainees, and rheumatology health professionals in Australia. At the time of the study, ARA membership comprised 377 rheumatologists, 112 advanced trainees in rheumatology, and 61 other health professionals (including nurses, physiotherapists, occupational therapists, podiatrists, scientists, and other health professionals with an interest in rheumatic disease). An email was sent to all 550 members of the ARA on 24 January 2020 and 28 April 2020 inviting them to participate in each round of the survey, respectively.

All ARA members were invited to participate in the second round of the survey regardless of their participation in the first round. A reminder email was sent 2 weeks after the initial invitation for each round of the survey. Each survey was closed 6 weeks after the initial email. All responses were anonymous.

Ethics approval was granted by the Central Adelaide Local Health Network Human Research Ethics Committee (CALHN reference number 12728).

### 2.2 Patient and public involvement

Patients and/or the public were not directly involved in the design, conduct, reporting, or dissemination plans of this research. Patients are involved in the production of the ALG, including guideline panel membership and authorship of recommendations.

### 2.3 Survey procedure

An online platform (SurveyMonkey, Momentive Inc.) was used to collect the data. The survey questions used in each round are included in Appendices S1 and S2.

The first survey round introduced the living guideline project and invited participants to nominate at least three (and up to 10) questions that they thought ought to be addressed in a guideline for the pharmacological management of RA. Potential questions were entered by participants as free text. Participants were encouraged to choose brief questions related to a specific aspect of their daily practice, rather than broad topics, and were provided with two example questions.

The complete set of questions submitted by participants in the first survey round was collated, duplicates were removed, and, where appropriate, similar or overlapping questions were merged. The remaining questions were further refined and collated to facilitate the final ranking survey. Submitted responses that contained only broad topics rather than clinical questions, those that did not relate to the pharmacological management of RA, and questions that were not directly related to clinical practice, were also removed. Where necessary, the wording of individual questions was edited for clarity, to incorporate overlapping themes, or to ensure that they were phrased as a clinical question.

The refined set of questions formed the basis for the second survey round. The questions were presented to participants in random order. Participants were instructed to choose 10 questions that they considered to be the highest priority and then rank them in order of importance from 10 (most important) to 1 (least important).

In addition, the first survey collected demographic information, including gender, number of years in clinical practice, and the location and type of practice (public hospital vs. private practice, urban vs. rural or regional). Participants were also asked about their current use of clinical practice guidelines, barriers to their use of guidelines, and whether they thought that Australian rheumatology guidelines were necessary.

### 2.4 Analysis

A final priority list was generated according to the sum of ranks. For each respondent, the highest‐ranked question received 10 points, decreasing by 1 point for each lower rank to a score of 1 point for the tenth‐ranked question. The sum of points allocated by all respondents for each question provided the total ranking score. The final set of questions in order from the highest to lowest total ranking score, the total score, and the number of respondents who included each question in their top 10 are reported. All other data are presented descriptively.

## 3 RESULTS

There were 115 (21%) and 78 (14%) responses to the first and second survey rounds respectively (see Table 1). 95% of participants in both survey rounds were rheumatologists, representing 29% and 20% of all Australian rheumatologists in the two surveys respectively. Only two rheumatology trainees participated in each round of the survey. The rheumatologists who participated in each round of the survey were representative of the Australian rheumatology workforce in terms of gender, years of experience, practice setting (private practice vs. public hospital), and practice location (urban vs. regional or rural).^13^ 43% of participants in the first survey and 51% of participants in the second survey were female, which is consistent with the proportion of female rheumatologists in Australia (47%). The gender distribution of respondents was also consistent with the Australian rheumatology workforce composition across all strata of age, practice type, and location. Hospital‐based rheumatologists and private practitioners were equally represented among survey respondents, and there was a broad range of clinical experience. Most participants reported working primarily in an urban setting, consistent with the Australian rheumatology workforce distribution.

**TABLE 1:** Characteristics of survey respondents and their guideline use.

	Survey 1 (*n* = 115)	Survey 2 (*n* = 78)
	*N* (%)	*N* (%)
Female	50 (43)	40 (51)
Discipline		
Rheumatologist	109 (95)	74 (95)
Rheumatology trainee	2 (2)	2 (3)
Rheumatology nurse	3 (3)	0 (0)
Other health professional	0 (0)	1 (1)
Other	1 (1)	1 (1)
Years involved in rheumatology		
0–5	16 (14)	9 (12)
6–10	21 (18)	18 (23)
11–20	25 (22)	18 (23)
>20	53 (46)	33 (42)
Location of practice		
Urban	85 (74)	59 (76)
Rural/Regional	14 (12)	9 (12)
Both urban and regional	14 (12)	8 (10)
Not practising	2 (2)	2 (3)
Are Australian rheumatology guidelines necessary?		
Yes	59 (51)	
No	22 (19)	
Unsure	34 (30)	
Use of rheumatology guidelines in usual practice		
Never	15 (13)	
Sometimes	68 (59)	
Often	32 (28)	
Guideline used most commonly (*n* = 99)		
EULAR	46 (46)	
ACR	36 (36)	
APLAR	0 (0)	
Other	17 (17)	
Barriers to guideline use (*n* = 77)		
Not representative of my patients	27 (35)	
Interrupt clinical interaction	27 (35)	
Personal preference	26 (34)	
Difficult to access	24 (31)	
Unnecessary with experience	23 (30)	
Interfere with autonomy	11 (14)	

A majority of respondents to the first survey (51%) indicated that they believed that Australian rheumatology guidelines are necessary, 19% indicated that they were not and a further 30% were unsure (Table 1). 87% reported using existing rheumatology clinical guidelines in their usual practice either “sometimes” (59%) or “often” (28%); the most commonly used guidelines were those produced by the European Alliance of Associations for Rheumatology (EULAR) (46%) and the American College of Rheumatology (ACR) (36%).

A variety of barriers to greater use of clinical guidelines were reported. The most common were concerns that guideline recommendations may not be applicable to individual patients (35% of respondents to this question), interrupt the clinical interaction (35%), and may be difficult to access (31%). The majority of respondents who currently use clinical guidelines, and a quarter of those who do not use guidelines, reported that they would use guidelines more frequently if they were integrated into their clinical practice software.

A total of 443 potential guideline questions were submitted by respondents to the first survey. After grouping and merging redundant and overlapping questions, 69 unique questions remained. This list of questions was further refined to remove questions that were outside the scope of the guideline (e.g., questions that were not related to pharmacotherapy). Where appropriate, questions with overlapping themes were further combined into a single question. For example, several questions relating to the use of disease‐modifying anti‐rheumatic drugs (DMARDs) in the setting of a specific comorbidity (e.g., liver disease or renal impairment) were combined into a broader question regarding DMARDs and comorbidity. The wording of the remaining questions was adjusted where necessary to retain the format of a clinical question. This resulted in a final set of 34 unique clinical questions which formed the basis for the second round of the survey. Table 2 lists these questions in order of their final ranking.

**TABLE 2:** Clinical questions ranked by survey participants.

Rank	Ranking score	Question	Number of respondents who included question in their top 10
			*N* (%)
1	287	What is the best approach to choosing a DMARD treatment strategy based on individual factors including disease severity, serological status, co‐morbidities, prognostic factors, and other predictors?	37 (47)
2	268	What is the best approach to management of RA in patients with important comorbidity (e.g., current or previous cancer, liver disease, lung disease, kidney disease, chronic infection, immunodeficiency)?	41 (53)
3	199	What is the best DMARD choice in patients with RA who have failed to respond to treatment with a first or multiple biologic or targeted synthetic DMARDs (b/tsDMARDs)?	31 (40)
4	194	How should DMARD therapy be used in women with RA who are pregnant or breastfeeding, and in women and men who are planning a pregnancy?	39 (50)
5	189	When and how should b/tsDMARDs and csDMARDs be tapered or discontinued in patients with RA who have responded well to treatment?	39 (50)
6	177	Which vaccinations should be offered to patients receiving treatment for RA, and when?	32 (41)
7	158	What is the best approach to the assessment and management of persistent or amplified pain in patients with RA?	29 (37)
8	152	What is the best approach to switching DMARD therapy in patients with RA?	28 (36)
9	143	What is the best approach to management of undifferentiated early inflammatory arthritis, including asymptomatic seropositive patients?	31 (40)
10	140	What is the best approach to the use of glucocorticoids in patients with RA?	28 (36)
11	137	What is the best DMARD choice in patients with RA who have failed to respond to, or are intolerant of, conventional synthetic DMARDs (csDMARDs)?	18 (23)
12	130	What are the best outcome measures and the treatment target in RA?	18 (23)
13	115	What is the best approach to monitoring for the adverse effects of csDMARDs?	21 (27)
14	108	How should DMARDs be used in the perioperative period?	22 (28)
15	95	What is the role of MTX monotherapy versus csDMARD combination therapy in patients with RA; and which method of combination of csDMARDs is best?	15 (19)
16	89	What is the best initial DMARD treatment in patients with RA who have not previously received DMARDs?	12 (15)
17	86	How should we optimize patient adherence to RA therapy?	15 (19)
18	82	When should b/tsDMARDs be used in combination with csDMARDs or with other b/tsDMARDs?	15 (19)
19	80	What is the optimal timing for the introduction of DMARDs in patients with early RA?	11 (14)
20	72	Which investigations should be performed before commencing csDMARDs or b/tsDMARDs?	11 (14)
20	72	What is the best treatment for extra‐articular manifestations of RA?	18 (23)
22	71	What is the best starting dose, target dose, and route of administration of methotrexate in patients with RA?	11 (14)
23	68	What is the role of imaging in aiding management decisions in RA?	16 (21)
23	68	What is the best DMARD therapy in elderly patients with RA?	14 (18)
25	64	How should we define remission in RA?	10 (13)
26	59	What is the best approach to the identification and management of cardiovascular risk in patients with RA?	16 (21)
27	43	What is the optimal frequency of clinical assessment in patients with RA?	11 (14)
28	33	How and when should biosimilar drugs be used in RA?	11 (14)
29	28	What is the best approach to monitoring for immunological adverse effects in patients treated with rituximab?	7 (9)
30	18	How should osteoporosis be managed in patients with RA?	7 (9)
31	17	What is the best approach to folate supplementation in patients treated with MTX for RA?	5 (6)
32	10	What is the role of NSAIDs in the management of RA?	4 (5)
33	8	What is the best approach to reducing the risk of retinopathy in patients with RA treated with hydroxychloroquine?	5 (6)
34	5	What is the role of dental health care in the management of RA	2 (3)

All 34 questions in the second survey were included in the top 10 by at least two respondents (Table 2). The question with the highest ranking score, selected in the top 10 by 47% of respondents, related to the best approach to individualizing the choice of DMARD in people with RA. Eighteen percent of all participants selected this question as the most important. The second highest‐ranked question, regarding the management of RA in the setting of important comorbidity, was selected in the top 10 by the highest proportion of respondents (53%) but had a lower total ranking score. Other questions ranked in the top five related to the best choice of DMARD in people with RA who have had an inadequate response to a biologic or targeted synthetic DMARD (b/tsDMARD), the use of DMARDs in women who are pregnant or breastfeeding, and the best approach to tapering or discontinuation of DMARDs.

## 4 DISCUSSION

Our study has identified and ranked 34 clinical questions regarding the pharmacological management of RA in order of importance to clinicians. This priority list is directly relevant to the development of a living guideline for several reasons. First, it enables the guideline recommendations to be developed in order of importance to users of the guidelines; less important questions may not need to be addressed at all, allowing resources to be allocated to topics identified as most important by guideline users. Second, guideline recommendations most suited to living methodology are those that are of high importance and for which there is existing uncertainty in the evidence base.^3^ In nominating the questions of most importance to them, survey respondents tended to identify questions for which the evidence base is currently sparse, and which are therefore potentially ideal for a living guideline if new evidence is expected to emerge. Third, the simple online survey methodology permits the process to be repeated intermittently in order to ensure that the priority list itself is a living entity.

Several of the highest‐ranked questions related to the best choice of DMARD therapy in people with RA. The introduction of b/tsDMARDs over the last two decades has led to a rapid expansion of therapeutic options and a consequent increase in the complexity of therapeutic decision‐making. The two highest‐ranked questions were both related to the individualization of treatment strategies, based either on individual predictors of response or major comorbidity. This may reflect the clinical observation that despite the overall improvement in RA management in recent years, individual disease responses may vary considerably, and reliable predictive biomarkers are lacking.^14^

Many other highly‐ranked questions are also related to clinical decisions that are specific to the modern era of rheumatology practice. For example, questions related to the best choice of DMARD in patients who have had an inadequate response to either conventional synthetic or b/tsDMARDs were ranked higher than those regarding initial treatment in patients with a new diagnosis of RA. In addition, decisions regarding the use of DMARDs during pregnancy, breastfeeding, or at the time of elective surgery may be more complex in the modern era and, as a result, questions on these topics were ranked highly. The question of how best to reduce or discontinue DMARDs in people with RA who have achieved a state of clinical remission was ranked in the top five and likely reflects the increasing proportion of patients for whom this is a possibility since the emergence of modern treatment strategies.

Several questions identified other important aspects of RA treatment, including the use of glucocorticoids, management of persistent pain, and monitoring for adverse effects of medications. Questions relating to more established interventions, including methotrexate monotherapy, hydroxychloroquine, NSAIDs, and supplemental folic acid, received much lower rankings, although even the lowest ranked questions (regarding dental care in RA and retinopathy associated with hydroxychloroquine use) were included in the top 10 priority list of at least two respondents.

There have been a few similar studies aimed at prioritizing questions for clinical guidelines in rheumatology. The 3e initiative used similar methodology to ascertain the questions of highest importance to clinicians as the basis for developing recommendations, but focused on a series of specific topics related to the management of inflammatory arthritis that were determined by a steering committee. Several of the questions identified in the 3e recommendations on the use of methotrexate in RA were also identified in our survey, including pre‐treatment investigations, starting dose, route of administration, use in combination with other DMARDs, and use during pregnancy, breastfeeding, and elective surgery.^15^

We have performed a similar prioritization exercise to inform the development of an Australian Living Guideline for the Management of Juvenile Idiopathic Arthritis (JIA).^16^ Several questions that are relevant to the management of both adult and juvenile inflammatory arthritis were identified in both surveys, including questions regarding DMARD tapering and discontinuation, switching DMARDs in those who have not responded adequately to treatment, the best investigations before instituting DMARDs, and the choice of outcome measures.^17^ Questions regarding pain management, vaccinations, and the best approach to the use of glucocorticoids were also identified in both studies.

Our study has also provided important insights into the current use of clinical practice guidelines by Australian rheumatologists, potential barriers to greater uptake of guidelines, and the questions of the highest importance in the clinic. There was strong support from respondents for the development of an Australian rheumatology guideline, with fewer than one in five considering an Australian guideline to be unnecessary. Prior to the recent development of the ALG, there have been no Australian guidelines on inflammatory arthritis in active development. The most recent local guideline on RA was published by the Royal Australian College of General Practitioners in 2009.^18^ As a result, despite 87% of our survey respondents reporting that they use guidelines at least some of the time, Australians have needed to use international guidelines, primarily those produced by EULAR and the ACR. While these are comprehensive and produced in accordance with rigorous methodology, neither the EULAR nor the ACR guidelines use living methods and they may not necessarily apply to the circumstances or needs of Australian clinicians and patients.

An additional barrier to guideline use identified in the first survey round was difficulty in accessing guidelines and interruption of the clinical interaction. A large proportion of survey respondents indicated that they would use guidelines more frequently if they were integrated into their electronic practice software, including those who do not currently use guidelines at all. The ALG is developed and hosted on an interactive web application (MAGICapp) that can be readily accessed at the point of care and includes relevant additional information including practical tips on the implementation of recommendations and patient decision aids.^19^

Optimization of uptake by clinicians is an important goal of guideline developers, in order to best achieve the overarching aims of clinical guidelines, namely better evidence‐based care and improved health outcomes. A number of factors have been identified that may improve the implementability of guidelines, including those related to both the content and the format of the guideline.^11^, ^20^ It is likely that guidelines that include potential users from the beginning of the development process will optimize the relevance of the guideline content and may further enhance implementability by increasing the credibility and awareness of the guideline among potential users.^11^

An important objective of the living evidence approach is to promote efficient synthesis and translation of existing and emerging evidence. Living systematic reviews are a key driver of efficiency by providing a single global summary of evidence for a particular question that is continuously updated in near real‐time as new evidence emerges. This obviates the need for guideline developers to generate de novo evidence syntheses on existing topics when new guidelines are developed, substantially reducing redundant work and waste of scarce financial and human resources.^21^ Ideally, guideline developers working in diverse geographic regions and practice contexts could contribute to the development and maintenance of a single living evidence synthesis for important clinical questions, which could in turn be used as a shared primary evidence source for guideline recommendations that are developed within the local context. Identification of the clinical questions that are most important to guideline users may allow limited resources to be directed to such a global evidence synthesis for the most important questions. Replication of the current survey in other healthcare settings may help to further define the questions most suited to international collaboration.

Our study has several strengths. Survey respondents were a broadly representative sample of Australian rheumatologists who will be the primary users of the ALG. The anonymous online ranking method allowed for questions to be submitted and ranked by all participants without influence from opinion leaders and free from any agenda or framework that might be influenced by external stakeholders including policymakers or the pharmaceutical industry. Importantly, although the questions have been identified by Australian rheumatologists in order to develop a guideline that will meet the specific needs of clinicians working in the Australian context, the list of questions is likely to be applicable to other healthcare settings and may help to inform prioritization in other countries.

There are some limitations to this study. The response rate (21% and 14% for the first and second survey respectively) was relatively small, however, response rate has been shown to be a poor predictor of response bias in survey research, particularly among relatively homogeneous professional groups.^22^, ^23^ The proportion of respondents in each of our survey rounds is broadly consistent with other similar surveys of professional groups, including response rates of 3.7% and 9.7% in a guideline prioritization survey undertaken among dermatologists.^24^ Given that respondents to our survey were representative of Australian rheumatologists in terms of gender, age, and type and location of practice, and over 400 questions were submitted in the first round of the survey, we consider our findings to be valid. Almost all participants were rheumatologists, despite membership of the ARA including rheumatology advanced trainees, allied health professionals, rheumatology nurses, and scientists. While rheumatologists are likely to be the primary users of the ALG, the relative lack of other participants may have limited the breadth or applicability of the findings. Further, we did not include healthcare consumers or other stakeholders (such as policymakers) in this survey. Inclusion of consumers, in particular, is an important component of guideline development and, although consumers have been included in all other steps in the development of the ALG, consumer participation in the prioritization survey is likely to have led to some differences in the priority list.^25^ We aim to include consumers in future iterations of our prioritization work.

Development of the ALG has begun, based in part on the high‐priority questions identified in this study pertaining to RA. A multidisciplinary guideline panel comprising rheumatologists, consumers, immunologists, allied health professionals, and general practitioners has developed several living recommendations relating to the use of DMARDs, opioids, and glucocorticoids in patients with RA, tapering of b/tsDMARDs, and the use of DMARDs in the perioperative period. The current set of living recommendations within the guideline has been approved by the Australian National Health and Medical Research Council (NHMRC). Further recommendations continue to be added and the existing recommendations are maintained in a living mode where appropriate. The priority list has been a key determinant of the choice of each recommendation developed so far, however, it has been our experience that other factors have also influenced the stepwise development of this living guideline, including the specificity of the clinical question, the volume of available evidence regarding each question, human and financial resources, and the emergence of new and unexpected high‐priority topics. The latter is exemplified by the urgent need for Australian recommendations on the use of COVID‐19 vaccines in patients on immunomodulatory medications for autoimmune and inflammatory rheumatic diseases, a question that could not have been foreseen when the first survey was performed in January 2020.^26^

## 5 CONCLUSION

Our study has identified 34 questions considered by Australian rheumatologists to be important in the management of RA, ranked in order of importance, that have informed the development of the world's first living guideline in rheumatology. Australian rheumatologists and rheumatology health professionals broadly support the development of Australian rheumatology guidelines. Involvement of guideline users from the beginning of the guideline development process allows scarce resources to be efficiently allocated to topics that are likely to be of the highest value in clinical practice, in order to enhance the use of guidelines at the point of care.

## AUTHOR CONTRIBUTIONS

SW and RB developed the study design and analyzed the data. SW wrote the first draft of the manuscript. All authors contributed to the performance of the study, interpretation of the data, and writing and final approval of the manuscript.

## FUNDING INFORMATION

This work is supported by the NHMRC Australia and New Zealand Musculoskeletal (ANZMUSC) Clinical Trials Network Centre of Research Excellence (2018‐2022, APP1134856). RB is supported by an Australian National Health and Medical Research Council (NHMRC) Investigator Fellowship (APP1194483) and SW is supported by an Australia and New Zealand Musculoskeletal (ANZMUSC) Clinical Trial Network Practitioner Fellowship and by a grant from The Hospital Research Foundation Group.

## CONFLICT OF INTEREST STATEMENT

The authors declare no conflicts of interest.

## Supporting information


Appendix S1

Appendix S2

## Data Availability

All data are available upon request.
