# Supplementary material for: Prioritization of clinical questions for the Australian Living Guideline for the Pharmacological Management of Inflammatory Arthritis
Source: Int J Rheum Dis. 2023 Sep 23;26(12):2410–8. doi: 10.1111/1756-185X.14926 (PMC10946502; doi:10.1111/1756-185X.14926)
Supplement: Supplementary file 1 — Appendix S1 [file APL-26-2410-s002.pdf]

ARA Membership Survey for RA Guidelines

The ARA is working with the Australia and New Zealand Musculoskeletal (ANZMUSC) Clinical Trials Network to develop a set of Australian guidelines for the management of rheumatoid arthritis. These will be ‘living’ guidelines, in which individual recommendations are updated in near real-time as new evidence emerges. Initially, a relatively small set of recommendations will be developed, but the living guideline format permits further recommendations to be added over time as resources permit. The first guideline will focus on the pharmacological management of rheumatoid arthritis.

In order to identify the topics of most importance to potential users of this guideline, we invite ARA members to participate in a prioritisation exercise. In this, the first round, we ask you to nominate at least 3 questions that you think ought to be addressed in a guideline for the pharmacological management of rheumatoid arthritis. A second and final survey round will ask participants to help rank the aggregated results of the current survey.

The survey will take less than 10 minutes to complete.

All responses are completely anonymous. Completion of the survey will imply that you consent to participate. You are free to withdraw at any time during the survey and none of your data will be collected.

The study has been approved by the Central Adelaide Local Health Network Human Research Ethics Committee. If you wish to speak to someone not directly involved in the study about your rights as a volunteer, or about the conduct of the study, you may also contact the CALHN HREC Chairperson, on 7117 2229 or 8222 6841.

CALHN reference number: 12728

ARA Membership Survey for RA Guidelines

* 1. What is your current role?

- ◯ Rheumatologist
- ◯ Rheumatology Advanced Trainee
- ◯ Rheumatology Nurse
- ◯ Other Rheumatology Health Professional
- ◯ Other (please specify)

* 2. Gender

- ◯ Female
- ◯ Male
- ◯ I'd rather not say

* 3. What is your primary place of practice?

- ◯ Hospital
- ◯ Private Practice
- ◯ N/A
- ◯ Other (please specify)

* 4. What is your primary location of practice?

- ◯ Urban
- ◯ Rural/Regional
- ◯ Both urban and regional
- ◯ N/A

5. Years involved in rheumatology:

- ◯ 0–5
- ◯ 6–10
- ◯ 11–20
- ◯ >20

* 6. Do you use an electronic health record in your usual practice?

- No
- Yes

If yes, which software?

* 7. Do you believe that Australian rheumatology guidelines are necessary?

- Yes
- No
- Unsure

If you answered 'no' or 'unsure', please add a comment about why you chose this answer.

8. Do you use any rheumatology guidelines in your usual practice?

- Never
- Sometimes
- Often

ARA Membership Survey for RA Guidelines

9. What prevents you from using guidelines? (tick all that apply)

- [ ] Personal preference
- [ ] Interrupt clinical interaction
- [ ] Not representative of my patients
- [ ] Difficult to access
- [ ] Interfere with practitioner autonomy
- [ ] Unnecessary because I have sufficient expertise

Other (please specify)

10. Would you use a guideline if it were integrated into your practice software?

- ○ Yes
- ○ No
- ○ Unsure

ANZMUSC
AUSTRALIA & NEW ZEALAND MUSCULOSKELETAL CLINICAL TRIALS NETWORK
NHMRC CENTRE OF RESEARCH EXCELLENCE

ARA Membership Survey for RA Guidelines

11. Which rheumatology guidelines do you use most commonly?

- ◯ ACR
- ◯ EULAR
- ◯ APLAR
- ◯ Therapeutic Guidelines
- ◯ Other (please specify)

12. What prevents you from using guidelines more often? (tick all that apply)

- ☐ Personal preference
- ☐ Not representative of my patients
- ☐ Interfere with practitioner autonomy
- ☐ Interrupt clinical interaction
- ☐ Difficult to access
- ☐ Unnecessary because I have sufficient expertise

Other (please specify)

13. Would you use guidelines more frequently if they were integrated into your practice software?

- ◯ Yes
- ◯ No
- ◯ Unsure

ARA Membership Survey for RA Guidelines

14. Which rheumatology guidelines do you use most commonly?

- ◯ ACR
- ◯ EULAR
- ◯ APLAR
- ◯ Therapeutic Guidelines
- ◯ Other (please specify)

[ ]

ARA Membership Survey for RA Guidelines

* 15. Nominate *at least 3* important questions for a guideline for the pharmacological management of rheumatoid arthritis. You may submit up to 10 questions. Please try to choose brief questions that relate to a specific aspect of your daily clinical practice, for example: “What is the best first-line DMARD in treatment-naive RA?” or "When should I taper DMARDs in RA patients in remission?".

1.

2.

3.

4.

5.

6.

7.

8.

9.

10.

16. Any other comments?
