# Supplementary material for: Prioritization of clinical questions for the Australian Living Guideline for the Pharmacological Management of Inflammatory Arthritis
Source: Int J Rheum Dis. 2023 Sep 23;26(12):2410–8. doi: 10.1111/1756-185X.14926 (PMC10946502; doi:10.1111/1756-185X.14926)
Supplement: Supplementary file 2 — Appendix S2 [file APL-26-2410-s001.pdf]

ARA Member Survey to Prioritise Guideline Questions

The ARA is working with the Australia and New Zealand Musculoskeletal (ANZMUSC) Clinical Trials Network to develop a set of Australian guidelines for the management of rheumatoid arthritis. These will be 'living' guidelines, in which individual recommendations are updated in near real-time as new evidence emerges. Initially, a relatively small set of recommendations will be developed, but the living guideline format permits further recommendations to be added over time as resources permit. The first guideline will focus on the pharmacological management of rheumatoid arthritis.

In order to identify the topics of most importance to potential users of this guideline, we invite all ARA members to participate in the second part of a prioritisation exercise. In the first round, members were asked to nominate at least 3 questions that they thought ought to be addressed in a guideline for the pharmacological management of rheumatoid arthritis. 443 separate questions were submitted by 114 respondents - these have been collated to remove redundancy and summarised into 34 unique questions.

In this, the second and final round of the survey, we invite you to help rank these questions by voting for those that you think are the highest priority. You will be asked to choose the 10 questions that you think are most important, and then rank these in order of importance.

You may participate in this ranking exercise whether or not you completed the first survey.

The survey will take less than 15 minutes to complete.

Following the rheumatoid arthritis ranking questions, there is also an opportunity to rank questions for a living guideline for the management of patients with rheumatic diseases in the setting of pandemic coronavirus. The questions have been developed in conjunction with the COVID-19 Global Rheumatology Alliance. This section of the survey is optional.

It is important to remember that the first part of this survey relates to the management of patients with rheumatoid arthritis, but the (optional) second part relates to the effect of COVID-19 on the management of all rheumatic diseases.

All responses are completely anonymous. Completion of the survey will imply that you consent to participate. You are free to withdraw at any time during the survey and none of your data will be collected.

The study has been approved by the Central Adelaide Local Health Network Human Research Ethics Committee. If you wish to speak to someone not directly involved in the study about your rights as a volunteer, or about the conduct of the study, you may also contact the CALHN HREC Chairperson, on 7117 2229 or 8222 6841.

CALHN reference number: 12728

ARA Member Survey to Prioritise Guideline Questions

* What is your current role?

- Rheumatologist
- Rheumatology Advanced Trainee
- Rheumatology Nurse
- Other Rheumatology Health Professional
- Other (please specify)

* Gender

- Female
- Male
- I'd rather not say

* What is your primary place of practice?

- Hospital
- Private Practice
- N/A
- Other (please specify)

* What is your primary location of practice?

- Urban
- Rural/Regional
- Both urban and regional
- N/A

* Years involved in rheumatology

- ◯ 0 - 5
- ◯ 6 - 10
- ◯ 11 - 20
- ◯ >20

ARA Member Survey to Prioritise Guideline Questions

* Choose the TEN most important questions for a living guideline for the pharmacological management of **rheumatoid arthritis**.

Please select exactly 10 questions. In the next section, you will be asked to rank your 10 choices in order of importance.

- [ ] When and how should b/tsDMARDs and csDMARDs be tapered or discontinued in patients with RA who have responded well to treatment?
- [ ] What is the best DMARD choice in patients with RA who have failed to respond to, or are intolerant of, conventional synthetic DMARDs (csDMARDs)?
- [ ] What is the best approach to the use of glucocorticoids in patients with RA?
- [ ] What is the best approach to management of RA in patients with important comorbidity (eg current or previous cancer, liver disease, lung disease, kidney disease, chronic infection, immunodeficiency)?
- [ ] What is the best initial DMARD treatment in patients with RA who have not previously received DMARDs?
- [ ] What is the role of MTX monotherapy versus csDMARD combination therapy in patients with RA; and which method of combination of csDMARDs is best?
- [ ] What is the best approach to choosing a DMARD treatment strategy based on individual factors including disease severity, serological status, co-morbidities, prognostic factors and other predictors?
- [ ] Which vaccinations should be offered to patients receiving treatment for RA, and when?
- [ ] What is the best DMARD choice in patients with RA who have failed to respond to treatment with a first or multiple biologic or targeted synthetic DMARDs (b/tsDMARDs)?
- [ ] What is the best approach to monitoring for the adverse effects of csDMARDs?
- [ ] How should DMARD therapy be used in women with RA who are pregnant or breastfeeding, and in women and men who are planning a pregnancy?
- [ ] What is the best starting dose, target dose, and route of administration of methotrexate in patients with RA?
- [ ] When should b/tsDMARDs be used in combination with csDMARDs or with other b/tsDMARDs?
- [ ] Which investigations should be performed before commencing csDMARDs or b/tsDMARDs?
- [ ] What are the best outcome measures and the treatment target in RA?
- [ ] What is the best approach to the assessement and management of persistent or amplified pain in patients with RA?
- [ ] What is the best approach to switching DMARD therapy in patients with RA?
- [ ] How should osteoporosis be managed in patients with RA?
- [ ] What is the best approach to reducing the risk of retinopathy in patients with RA treated with hydroxychloroquine?

- [ ] What is the optimal timing for the introduction of DMARDs in patients with early RA?
- [ ] What is the optimal frequency of clinical assessment in patients with RA?
- [ ] What is the best approach to the identification and management of cardiovascular risk in patients with RA?
- [ ] What is the role of of imaging in aiding management decisions in RA?
- [ ] How should DMARDs be used in the peri-operative period?
- [ ] What is the best approach to monitoring for immunological adverse effects in patients treated with rituximab?
- [ ] How should we optimise patient adherence to RA therapy?
- [ ] What is the best approach to folate supplementation in patients treated with MTX for RA?
- [ ] What is the best DMARD therapy in elderly patients with RA?
- [ ] How should we define remission in RA?
- [ ] How and when should biosimilar drugs be used in RA?
- [ ] What is the best approach to management of undifferentiated early inflammatory arthritis, including asymptomatic seropositive patients?
- [ ] What is the role of dental health care in the management of RA
- [ ] What is the role of NSAIDs in the management of RA?
- [ ] What is the best treatment for extra-articular manifestations of RA?

ARA Member Survey to Prioritise Guideline Questions

* Please rank the 10 questions you have chosen (from MOST important to LEAST important)

You may click on the box beside each question to enter a ranking score from 1 (most important) to 10 (least important), or move each question up and down by clicking on the horizontal bars beside each question to generate a list from most important (at the top) to least important (at the bottom).

- When and how should b/tsDMARDs and csDMARDs be tapered or discontinued in patients with RA who have responded well to treatment?
- What is the best DMARD choice in patients with RA who have failed to respond to, or are intolerant of, conventional synthetic DMARDs (csDMARDs)?
- What is the best approach to the use of glucocorticoids in patients with RA?
- What is the best approach to management of RA in patients with important comorbidity (eg current or previous cancer, liver disease, lung disease, kidney disease, chronic infection, immunodeficiency)?
- What is the best initial DMARD treatment in patients with RA who have not previously received DMARDs?
- What is the role of MTX monotherapy versus csDMARD combination therapy in patients with RA; and which method of combination of csDMARDs is best?
- What is the best approach to choosing a DMARD treatment strategy based on individual factors including disease severity, serological status, co-morbidities, prognostic factors and other predictors?
- Which vaccinations should be offered to patients receiving treatment for RA, and when?
- What is the best DMARD choice in patients with RA who have failed to respond to treatment with a first or multiple biologic or targeted synthetic DMARDs (b/tsDMARDs)?
- What is the best approach to monitoring for the adverse effects of csDMARDs?
- How should DMARD therapy be used in women with RA who are pregnant or breastfeeding, and in women and men who are planning a pregnancy?
- What is the best starting dose, target dose, and route of administration of methotrexate in patients with RA?

When should b/tsDMARDs be used in combination with csDMARDs or with other b/tsDMARDs?

Which investigations should be performed before commencing csDMARDs or b/tsDMARDs?

What are the best outcome measures and the treatment target in RA?

What is the best approach to the assessement and management of persistent or amplified pain in patients with RA?

What is the best approach to switching DMARD therapy in patients with RA?

How should osteoporosis be managed in patients with RA?

What is the best approach to reducing the risk of retinopathy in patients with RA treated with hydroxychloroquine?

What is the optimal timing for the introduction of DMARDs in patients with early RA?

What is the optimal frequency of clinical assessment in patients with RA?

What is the best approach to the identification and management of cardiovascular risk in patients with RA?

What is the role of of imaging in aiding management decisions in RA?

How should DMARDs be used in the peri-operative period?

What is the best approach to monitoring for immunological adverse effects in patients treated with rituximab?

How should we optimise patient adherence to RA therapy?

What is the best approach to folate supplementation in patients treated with MTX for RA?

What is the best DMARD therapy in elderly patients with RA?

How should we define remission in RA?

How and when should biosimilar drugs be used in RA?

What is the best approach to management of undifferentiated early inflammatory arthritis, including asymptomatic seropositive patients?

What is the role of dental health care in the management of RA

What is the role of NSAIDs in the management of RA?

What is the best treatment for extra-articular manifestations of RA?

* Would you like to also rank questions for living recommendations for the management of patients with rheumatic diseases during the COVID-19 pandemic?

- ◯ Yes
- ◯ No
